# A mass spectrometric method for in-depth profiling of phosphoinositide regioisomers and their disease-associated regulation

**DOI:** 10.1038/s41467-021-27648-z

**Published:** 2022-01-10

**Authors:** Shin Morioka, Hiroki Nakanishi, Toshiyoshi Yamamoto, Junya Hasegawa, Emi Tokuda, Tomoya Hikita, Tomoko Sakihara, Yuuki Kugii, Chitose Oneyama, Masakazu Yamazaki, Akira Suzuki, Junko Sasaki, Takehiko Sasaki

**Affiliations:** 1 Department of Biochemical Pathophysiology, Medical Research Institute, Tokyo Medical and Dental University, Tokyo, 113-8510 Japan; 2 Department of Lipid Biology, Graduate School of Medical and Dental Sciences, Tokyo Medical and Dental University, Tokyo, 113-8510 Japan; 3 Research Center for Biosignal, Akita University, Akita, 010-8543 Japan; 4 Division of Cancer Cell Regulation, Aichi Cancer Center Research Institute, Chikusa-ku, Nagoya, 464-8681 Japan; 5 Department of Cell Biology and Morphology, Akita University Graduate School of Medicine, Akita, 010-8543 Japan; 6 Division of Molecular and Cellular Biology, Graduate School of Medicine, Kobe University, Kobe, 650-0017 Japan; 7 Department of Life Science and Technology, Graduate School of Medical and Dental Sciences, Tokyo Medical and Dental University, Tokyo, 113-8510 Japan

**Keywords:** Phospholipids, Mass spectrometry, Chromatography, Lipidomics

## Abstract

Phosphoinositides are a family of membrane lipids essential for many biological and pathological processes. Due to the existence of multiple phosphoinositide regioisomers and their low intracellular concentrations, profiling these lipids and linking a specific acyl variant to a change in biological state have been difficult. To enable the comprehensive analysis of phosphoinositide phosphorylation status and acyl chain identity, we develop PRMC-MS (Phosphoinositide Regioisomer Measurement by Chiral column chromatography and Mass Spectrometry). Using this method, we reveal a severe skewing in acyl chains in phosphoinositides in *Pten*-deficient prostate cancer tissues, extracellular mobilization of phosphoinositides upon expression of oncogenic PIK3CA, and a unique profile for exosomal phosphoinositides. Thus, our approach allows characterizing the dynamics of phosphoinositide acyl variants in intracellular and extracellular milieus.

## Abstract (web summary)

Different phosphoinositide isomers are involved in a variety of physiological and pathological processes. Here, the authors combine chiral column chromatography and mass spectrometry to measure phosphoinositide regioisomers, revealing their dynamic changes in intra- and extracellular cancer cell milieus.

## Introduction

**Fig. 1:**
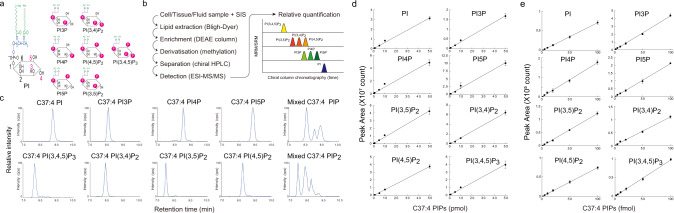
Phosphoinositide regioisomer measurement by PRMC-MS. **a** Schematic structures of the eight phosphoinositide classes. Note that phosphatidylinositol mono- and bisphosphate (PIP and PIP_2_) classes each contain three regioisomers. **b** Scheme depicting the general workflow of PRMC-MS. SIS, surrogate internal standard lipids. **c** Mass spectra from PRMC-MS analyses of C37:4 (1-heptadecanoyl-2-eicosatetraenoyl) standard chemicals representing each of the eight phosphoinositide classes. Mixed, a mixture of PIP or PIP_2_ regioisomers. **d**, **e** Linear regression analyses of peak area vs pmol (**d**) and fmol (**e**) concentrations of the indicated phosphoinositides. Amounts of standard phosphoinositides analyzed were 0.002, 0.005, 0.01, 0.02, 0.05, 0.1, 0.2, 0.5, 1, 5, 10, 50 pmol in **d** and 2, 5, 10, 20, 50, 100 fmol in **e** (see also Supplementary Table 1). The C37:4 (1-heptadecanoyl-2-eicosatetraenoyl) molecular species of each phosphoinositide class was used. Data were the mean ± SD (*n* = 4 technical replicates). Source data are provided as a Source Data file.

Phosphoinositides comprise one of the most functionally versatile membrane lipid families involved in human health and disease^1,2^. The base structure of all phosphoinositides contains phosphatidylinositol (PI), which is made up of an inositol head group and two long-chain fatty acids linked to a glycerol backbone (Fig. 1a). Combinatorial phosphorylation of residues in the PI head group gives rise to seven other phosphoinositide classes, namely PI(3)P, PI(4)P, PI(5)P, PI(3,4)P_2_, PI(3,5)P_2_, PI(4,5)P_2_, and PI(3,4,5)P_3_. These lipids spatiotemporally control the activities of many proteins possessing phosphoinositide-binding motifs. These motifs can bind to various phosphoinositides with differing affinities to regulate numerous physiological processes in cells^3^.

Genetic alterations in phosphoinositide-metabolizing enzymes, including kinases, phosphatases, lipases, and acyltransferases, have been implicated in the pathogenesis of various diseases^4,5^. However, it is unclear whether or how these genetic changes alter phosphoinositide configurations; that is, it is not known if the composition of various phosphoinositide classes and their acyl variants differ in the cells or tissues of diseased individuals compared to healthy individuals. Part of the challenge in addressing this issue lies in the current difficulties in measuring specific phosphoinositide acyl variants.

Measurements of cellular phosphoinositides have traditionally been carried out by anion-exchange HPLC of lipid extracts prepared from cultured cells that were metabolically labeled with [^3^H]inositol and/or [^32^P]phosphorus^6^. Although this method effectively quantifies the relative levels of the eight phosphoinositide classes, it cannot easily be applied to clinical samples or pathological samples from experimental animals. In addition, this approach cannot provide any information on differences in the acyl chains of phosphoinositide species because the hydrophobic moiety has to be removed before separation by anion-exchange chromatography. Recently, Clark et al. devised a significant improvement in phosphoinositide measurement by combining reverse-phase HPLC with electrospray ionization-tandem mass spectrometry (ESI-MS/MS)^7^. This method enables the quantification of phosphoinositide molecular species in a spectrum of biological samples, including those not amenable to radioisotope labeling. Ghosh et al. have also described a mass spectrometry method based on the use of recombinant specific lipid-kinases to in vitro phosphorylate PI3P and PI5P into PI(3,5)P_2_ and PI(4,5)P_2_, respectively^8^. However, it still remains a hurdle to simultaneously quantitate the acyl variants of all individual regioisomers of PI-monophosphate (PI3P/PI4P/PI5P) and -bisphosphate [PI(3,4)P_2_/PI(3,5)P_2_/PI(4,5)P_2_] as well as PI and PI(3,4,5)P_3_ in a biological sample^9^. A method that permits comprehensive measurement of the entire set of phosphoinositides would advance our understanding of how all these lipids are dynamically engaged to control cell functions.

Here we report a method for Phosphoinositide Regioisomer Measurement by Chiral column chromatography and Mass Spectrometry (PRMC-MS) that solves the above problem and can be applied to many types of biological samples. PRMC-MS reveals that phosphoinositide acyl variants are differentially accumulated in normal mouse prostate and *Pten*-deficient prostate cancer tissues and further identifies extracellular mobilization of the phosphoinositide variants.

## Results

### An HPLC-MS-based method to measure the eight phosphoinositide classes at the acyl variant level

The general workflow of our PRMC-MS method to measure phosphoinositide acyl variants (defined by the number of carbons and the degree of unsaturation in the hydrocarbon chains) is depicted in Fig. 1b. We devised this method to circumvent two confounding issues: (1) The anion-exchange column chromatography approach previously introduced for the separation of glycerophosphoinositol phosphates (the deacylated products of phosphoinositides) cannot be easily adapted for ESI-MS/MS due to the requirement for a non-volatile phosphate buffer. (2) Reverse-phase columns do not discriminate among phosphoinositide regioisomers. We, therefore, set out to adapt chiral column chromatography and took advantage of the mobile phases of acetonitrile and methanol. Synthetic phosphoinositides (heptadecanoyl-arachidonyl; C37:4 molecular species) were used as standard analytes whose phosphate groups had been methylated by a reaction with trimethylsilyl (TMS) diazomethane^7^. The derivatized phosphoinositides were mixed in various combinations, separated on a stationary phase where a chiral selector was immobilized to silica gel matrices, and detected with an in-line triple quadrupole mass spectrometer using the “multiple reaction monitoring” (MRM) technique^10^. During trial-and-error testing of chromatography columns, the chiral selector cellulose tris(3,5-dichlorophenylcarbamate) was found to successfully separate the three PI-monophosphate (PIP) and three PI-bisphosphate (PIP_2_) regioisomers (Fig. 1c and Supplementary Fig. 1). PI and PI-trisphosphate [PI(3,4,5)P_3_] could also be analyzed in the same run using different MRM transitions. Thus, our method can simultaneously measure all eight classes of phosphoinositides in a single sample. Repeatable quantification was confirmed by 50 successive measurements of C37:4 phosphoinositide standards with a coefficient of variation (CV) values of 4.9–12.0% (Supplementary Table 1). Good linear correlations were obtained between the ion-current peak areas and chemical amounts ranging from sub-fmol to 50 pmol of the phosphoinositide standards (Fig. 1d, e and Supplementary Table 1). These data establish that our PRMC-MS approach has a wide dynamic range and a low detection limit for each phosphoinositide class.

### Application of PRMC-MS to intracellular phosphoinositide measurement

Next, we examined whether our PRMC-MS method was able to measure phosphoinositides in lipid extracts prepared from HEK293T cells. Considering the minor proportion of phosphoinositides to total phospholipids in cellular lipid extracts, an acidic lipid-enriched fraction was obtained using an anion-exchange column prior to the methylation reaction to prevent ion suppression in mass spectrometry. PRMC-MS successfully distinguished three regioisomers each of PIP and PIP_2_ in lipid extracts of HEK293T cells, and pre-concentration by passage over DEAE Sepharose enhanced the peak intensities of all phosphoinositides (Supplementary Fig. 2a). The yields [peak area with pre-concentration divided by peak area without pre-concentration (triplicate average)] were 20.2% (PI), 92.2% (PI3P), 81.9% (PI4P), 89.6% (PI5P), 167% (PI(3,4)P_2_), 128% (PI(3,5)P_2_), 134% (PI(4,5)P_2_), and 113% (PI(3,4,5)P_3_) when determined using synthetic C37:4 phosphoinositides. This enrichment procedure was especially effective for measuring minor phosphoinositide classes such as PI3P, PI5P, PI(3,4)P_2_, PI(3,5)P_2_, and PI(3,4,5)P_3_, as well as some minor acyl variants of PI, PI4P, and PI(4,5)P_2_, although it failed to steadily detect previously reported PIP and PIP_2_ variants containing highly polyunsaturated acyl chains (e.g., 40:7, 36:8, 40:9, and 42:10)^11^. Pre-concentration was also helpful in reducing the maintenance/cleaning of the mass spectrometer because the amount of lipid loaded could be kept to a minimum.

**Fig. 2:**
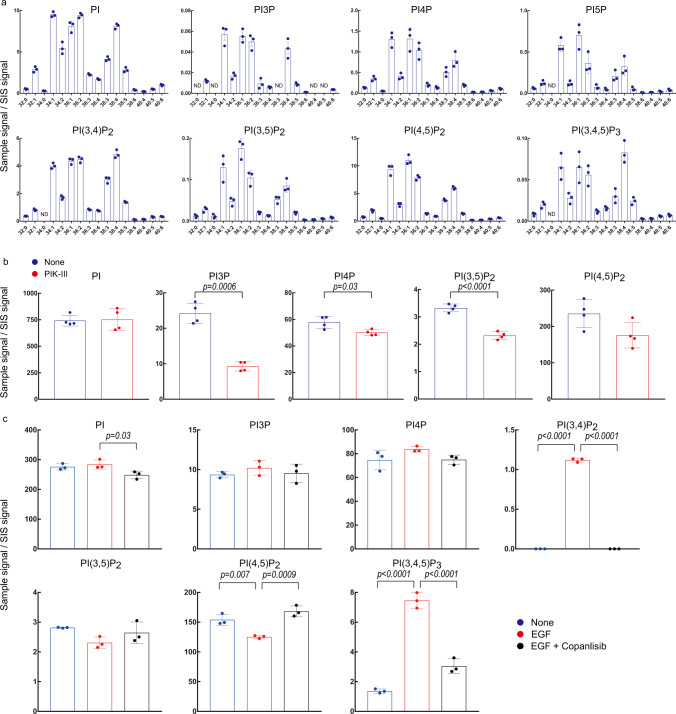
Application of PRMC-MS to phosphoinositide measurement in cell lines. **a** Levels of the indicated phosphoinositide acyl variants in HEK293T cells. PI, PI3P, PI4P, PI(3,5)P_2_, PI(4,5)P_2_, and PI(3,4,5)P_3_ were assessed in mock-transfected cells. PI5P was assessed in cells overexpressing IpgD. PI(3,4)P_2_ was assessed in non-transfected cells treated with 1 mM H_2_O_2_ for 5 min. Data were the mean ± SD (*n* = 3 biologically independent samples). ND not determined. **b** Phosphoinositide measurements in HeLa cells treated with vehicle (control) or 5 µM PIK-III for 2 h. PI5P, PI(3,4)P_2_, and PI(3,4,5)P_3_ were not detected. Data were the mean ± SD of total acyl variants of the indicated phosphoinositide classes (*n* = 4 biologically independent samples), and *p* values by two-sided Welch’s *t*-test are shown. **c** Phosphoinositide measurements in PC3 cells that were left untreated (control), or treated with vehicle or 100 nM copanlisib for 30 min prior to stimulation with 10 ng/ml EGF. PI5P was not detected. Data were the mean ± SD of total acyl variants of the indicated phosphoinositide classes (*n* = 3 biologically independent samples). The significance was analyzed using a two-sided Dunnett’s *t*-test. Source data are provided as a Source Data file.

Supplementary Table 2 lists the MRM transitions (pairs of *m/z* values of precursor ions and fragment/diacylglycerol ions) used for the identification and quantification of each phosphoinositide acyl variant. Endogenous concentrations of the acyl variants in each class were estimated based on those of the class-matched C37:4 phosphoinositides (surrogate internal standards; SIS) added to the cell pellets before lipid extraction, allowing the relative quantification of intracellular acyl variants. Practically, the chromatographic peak area of an endogenous phosphoinositide was divided by the area of the class-matched SIS (Supplementary Fig. 3). As shown in Fig. 2a, PI, PI3P, PI4P, PI(3,5)P_2_, PI(4,5)P_2_, and PI(3,4,5)P_3_ were readily detected in resting HEK293T cells. Approximately linear correlations were obtained between cell numbers and the amounts of the phosphoinositide acyl variants identified (Supplementary Fig. 4 and Supplementary Table 3). We did not find the peaks corresponding to any acyl variants of PI(5)P and PI(3,4)P_2_ in resting HEK293T cells probably because these lipids were present below the detection limit of PRMC-MS under normal culture conditions. To test whether PRMC-MS can detect cellular PI(5)P, we analyzed HEK293T cells engineered to overexpress IpgD [a *Shigella flexneri* virulence factor that converts PI(4,5)P_2_ to PI5P^12^]. In parallel, to detect intracellular PI(3,4)P_2_, we treated non-transfected HEK293T cells with hydrogen peroxide (H_2_O_2_)^13^. We found that IpgD expression led to an increase in PI5P, whereas H_2_O_2_ treatment increased PI(3,4)P_2_ (Fig. 2a). Thus, PRMC-MS allows the simultaneous measurement of all 8 phosphoinositide classes at the acyl variant level in cell extracts.

Vps34 and the class II PI3Ks produce PI3P, and various PI3P pools are proposed to be involved in distinct cellular processes, including autophagic programs^14^. PIK-III is a potent and selective Vps34 inhibitor that binds a unique hydrophobic pocket not present in class I/II PI3Ks^15^. PIK-III decreased PI3P by ~40% in HeLa cells, indicating that PRMC-MS can differentiate between Vps34-dependent and -independent PI3P pools within cells (Fig. 2b). In addition, there were smaller but statistically significant decreases in PI(3,5)P_2_ (a phosphorylated derivative of PI3P) and PI4P levels between control and PIK-III treated cells. Similarly, previous work has shown that epidermal growth factor (EGF) induces the accumulation of PI(3,4)P_2_ and PI(3,4,5)P_3_ in a class I PI3K-dependent manner^16^. Using PRMC-MS to examine EGF-treated PC3 cells, we found that PI(3,4)P_2_ and PI(3,4,5)P_3_ were increased while PI(4,5)P_2_ (the primary substrate of class I PI3Ks) was decreased (Fig. 2c). We also confirmed that these responses were abolished by the pan-class I PI3K inhibitor copanlisib^17^. Thus, PRMC-MS is exquisitely sensitive and highly useful for the detection of small but important changes in intracellular phosphoinositide levels in response to environmental cues.

### Distorted phosphoinositide profile in prostate cancer tissue from *Pten*-deficient mice

**Fig. 3:**
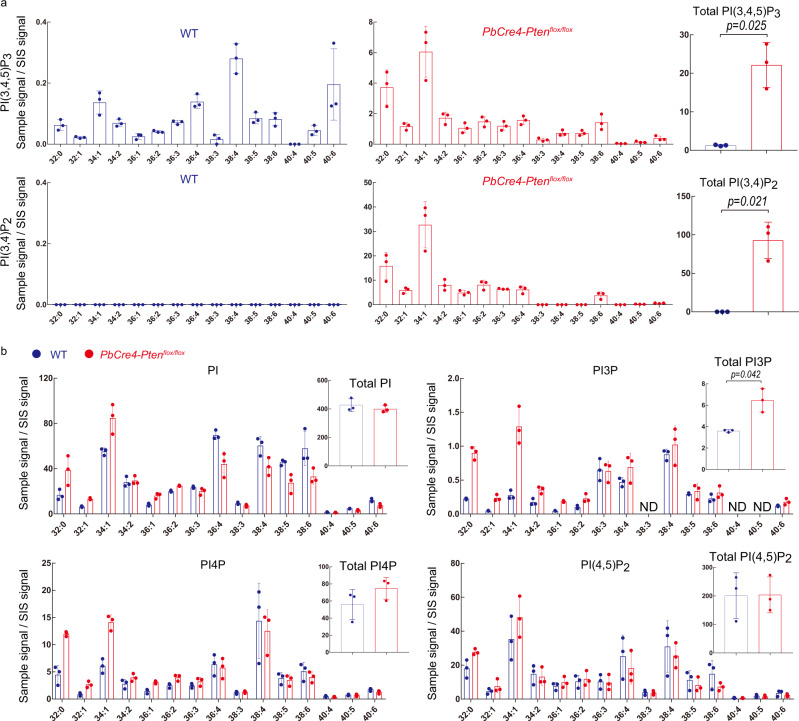
Application of PRMC-MS to phosphoinositide measurement in mouse tissue. **a** Left and middle panels: Quantification of the indicated PI(3,4,5)P_3_ and PI(3,4)P_2_ acyl variants in prostate tissues from WT and prostate-specific *Pten*-deficient (*PbCre4-Pten*^*flox/flox*^) mice at 12 weeks of age (*n* = 3 independent animals/group). The altered acyl chain profile in *Pten*-deficient cancerous tissues was confirmed in five independent experiments. Right panel: Total of the PI(3,4)P_2_ and PI(3,4,5)P_3_ acyl variants in the tissues in the left and middle panels. **b** Measurements of the indicated acyl variants and total PI, PI3P, PI4P, and PI(4,5)P_2_ in the prostate tissues from the WT (blue) and *PbCre4-Pten*^*flox/flox*^ (red) mice in (**a**) (12 weeks of age, *n* = 3). For all panels, data were the mean ± SD (*n* = 3 independent animals). ND not determined. The significance between WT and *PbCre4-Pten*^*flox/flox*^ mice was analyzed using a two-sided Welch’s *t*-test. Source data are provided as a Source Data file.

In vivo measurement of phosphoinositides has been limited due to the necessity of radioisotope labeling of animals, which is hazardous and labor-intensive. We exploited a cancer mouse model to test our PRMC-MS method in tissue samples. The phosphatase PTEN, whose primary substrate is PI(3,4,5)P_3_, is one of the most frequently altered tumor suppressors across a variety of cancer types^18^. We used PRMC-MS to analyze the phosphoinositide profiles of prostate tissues isolated from healthy wild-type (WT) C57BL/6 mice and those from prostate tumor-bearing *Pten*-deficient (*PbCre4-Pten*^*flox/flox*^;^19,20^) mice (Fig. 3). Consistent with a previous study employing HPLC-MS analysis and immunohistochemistry^16^, levels of PI(3,4,5)P_3_ and PI(3,4)P_2_ were heightened in in vivo samples of the prostates of *Pten*-deficient mice. In addition, PI3P was increased while PI4P remained unchanged in *Pten*-deficient prostates. Intriguingly, we observed a dramatic skewing in the acyl variant composition of PI(3,4,5)P_3_ in the *Pten*-deficient prostates. For instance, levels of C32:0 and C34:1 PI(3,4,5)P_3_ were increased ~50-fold in the tumor samples compared to controls, whereas C38:4 PI(3,4,5)P_3_ (the most abundant acyl variant in normal prostate) was almost unchanged. The fatty acyl profile of PI(3,4)P_2_ in cancerous prostate tissue resembled that of PI(3,4,5)P_3_, supporting the previously described notion that PI(3,4)P_2_ in tumors is produced mainly through 5′-dephosphorylation of PI(3,4,5)P_3_^16^. These data demonstrate that not only the quantity but also the composition of PI(3,4)P_2_ and PI(3,4,5)P_3_ are disturbed in *Pten*-deficient cancer tissue. Our results further suggest that the detailed profiling of phosphoinositide acyl variants may reveal molecular signatures useful for cancer stratification.

### Extracellular mobilization of phosphoinositides

**Fig. 4:**
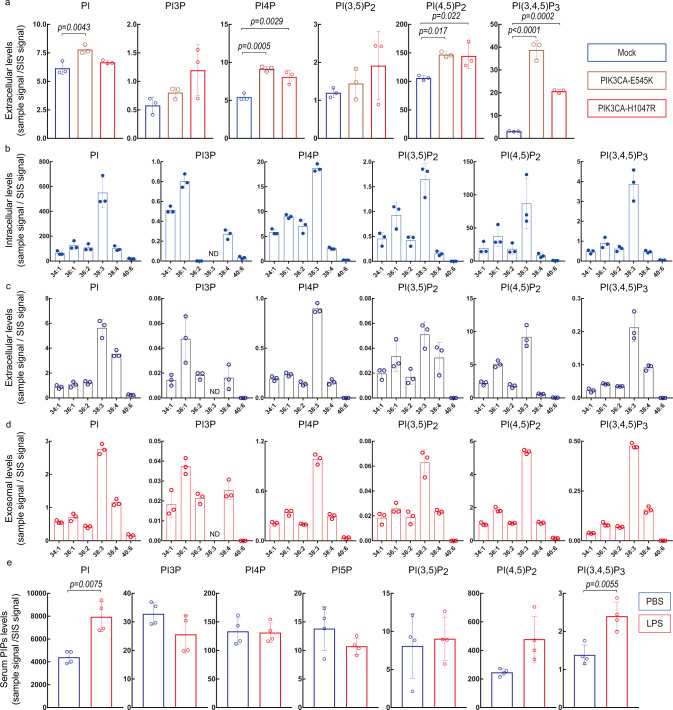
Application of PRMC-MS to phosphoinositide measurement in liquid samples. **a** Quantification of the indicated phosphoinositides in CM of HEK293T cells transfected with either control expression vector (Mock) or vector expressing either mutant E545K or H1047R PIK3CA. CM was collected at 24 h post-transfection. Data were the mean ± SD (*n* = 3 biologically independent samples) and representative of three independent experiments. Statistical analysis was performed using a two-sided Dunnett’s *t*-test. **b**–**d** Quantification of the indicated phosphoinositide acyl variants in **b** cell pellets, **c** CM, and **d** exosomes derived from LNCap cell cultures. PI5P and PI(3,4)P_2_ were not detected. Data were the mean ± SD (*n* = 3 biologically independent samples). **e** Quantification of the indicated phosphoinositides in plasma from WT mice that were intraperitoneally injected with either vehicle (PBS) or 50 mg/ml lipopolysaccharide (LPS) and analyzed after 2 h. PI(3,4)P_2_ was not detected in either case. Data were the mean ± SD of total acyl variants of the indicated phosphoinositide classes (*n* = 4 biologically independent samples). Statistical analysis was performed using two-sided unpaired Welch’s *t*-test. Source data are provided as a Source Data file.

The question of the existence of “extracellular” phosphoinositides has also been difficult to address using existing protocols, and so we investigated whether our PRMC-MS method could be extended to address this issue. We successfully detected PI, PI3P, PI4P, PI(3,5)P_2_, PI(4,5)P_2_, and PI(3,4,5)P_3_ in culture supernatants (conditioned medium; CM) of resting HEK293T cells (Fig. 4a). To determine the effect of oncogenic phosphoinositide 3-kinase expression on extracellular phosphoinositides, we examined HEK293T cells overexpressing various mutant PIK3CA proteins. *PIK3CA* gain-of-function mutations frequently occur at two hotspots: one in the helical domain (E545K) and one in the kinase domain (H1047R)^4^. We found that ectopic expression of these mutant PIK3CA molecules caused enormous increases in extracellular PI(3,4,5)P_3_, with smaller or no increases in other phosphoinositide classes (Fig. 4a). The presence of extracellular phosphoinositides was not unique to HEK293T cell cultures as these lipids were detected in CM of all cell lines tested, including LNCap cells (Fig. 4b–d).

Because phosphoinositides were detected in culture supernatants passed through 0.45 μm pores, it was unlikely that these lipids were derived from floating dead cells. To gain insight into the origin of these extracellular phosphoinositides, we isolated exosomes (extracellular vesicles of 50–150 nm in size) from LNCap culture supernatants. PRMC-MS revealed that these exosomes did indeed contain substantial amounts of phosphoinositides that accounted for about 10% of total phosphoinositides in CM (Fig. 4d). The acyl variant compositions of phosphoinositides in exosomes and CM were similar to those in intracellular samples (Fig. 4c,d and Supplementary Fig. 5).

The above findings in cultured cells prompted us to examine extracellular phosphoinositides in vivo. Blood samples were collected from WT mice that had been injected with either phosphate-buffered saline (PBS; control) or bacterial lipopolysaccharide (LPS). In control mice, all phosphoinositide classes except PI(3,4)P_2_ were detected in the plasma (Fig. 4e). LPS treatment increased plasma levels of PI and PI(3,4,5)P_3_. These data establish that PRMC-MS can be applied to blood samples to track phosphoinositide signatures potentially related to inflammatory disease states. In the future, it will be interesting to investigate the specific biological activity of the extracellular form of each class of phosphoinositide.

## Discussion

The PRMC-MS approach described here enables the comprehensive analysis of phosphoinositide acyl variants in various types of biological samples, including cultured cells, CM, exosomes, isolated tissues, and blood. We have shown that PRMC-MS can be used to highlight previously unrecognized disturbances of phosphoinositide fatty acyl profiles in cancerous tissue and to monitor the extracellular mobilization of phosphoinositides.

The physiological importance of molecularly heterogeneous acyl chain compositions of phosphoinositides in a cell has long been an enigma. Further studies should determine the cancer cell characteristics affected by the observed skewing in phosphoinositide acyl chains in the *Pten*-deficient prostate. An attractive hypothesis is that different acyl variants confer different protein-binding properties due to the influence of the hydrophobic moiety on the interaction of the lipid with proteins at the cell membrane. Such interactions could potentially activate a signaling pathway that favors cancer cell growth and survival and emerge as a target for the therapy of cancer. We previously analyzed phosphoinositide acyl variants in prostate cancer tissues from 16 surgical patients using reverse-phase chromatography-tandem mass spectrometry; however, we did not find a similar acyl chain distortion as in *Pten*-deficient mouse samples. Investigations using a larger number and more diverse types of human cancers should be conducted to elucidate the pathological relevance of the fluctuations in each acyl variant as well as their interaction with changes in oncogenes and tumor suppressor genes such as *PIK3CA*, *K-*, *H-RAS*, and *PTEN*.

Another finding of our study is its demonstration of the existence of extracellular phosphoinositides. Extracellular phosphoinositides were mobilized, at least in part, in the form of the exosomes. These results indicate that cells actively secrete phosphoinositides, which may in turn act on neighboring cells. Our finding that cellular activation by oncogenic PI3K mutant expression or LPS administration changes the extracellular phosphoinositide profile raises the possibility that autocrine/paracrine secretion of phosphoinositides may play a role in the pathogenesis of cancers and inflammatory diseases. Thus, PRMC-MS may pave the way for a field of study on the pathophysiological functions of phosphoinositides in the extracellular environment.

While this study was under review, Li and Lämmerhofer reported a method for the measurement of phosphoinositide regioisomers^21^. It also took advantage of chiral chromatography for regioisomer separation as PRMC-MS, though the chiral selector used was cellulose tris(3,5-dimethylphenylcarbamate). In addition, a different data acquisition method, sequential window acquisition of all theoretical mass spectra (SWATH), was adopted in the method.

A growing body of evidence demonstrates that alterations to genes encoding phosphoinositide kinases and phosphatases (which shape phosphorylation patterns) and lysophospholipid acyltransferases (which shape the acyl chains) are associated with cellular dysfunction and disease^9,22^. The use of PRMC-MS to evaluate phosphoinositide signatures at the acyl variant level in tissue and liquid biopsies in a particular disease context may reveal biomarkers suitable for a wide variety of clinical applications. Such applications may then facilitate rational drug development strategies based on the devising of a therapeutic agent that pinpoints a specific pathogenic phosphoinositide acyl variant.

## Methods

### Lipids and reagents

1-heptadecanoyl-2- (5Z, 8Z, 11Z, 14Z-eicosatetraenoyl)-*sn*-glycero-3-phospho-(1-myo-inositol-3,4,5-trisphosphate) [17:0/20:4 PI(3,4,5)P_3_], 17:0/20:4 PI(3,4)P_2_, 17:0/20:4 PI(3,5)P_2_, 17:0/20:4 PI(4,5)P_2_, 17:0/20:4 PI3P, 17:0/20:4 PI4P, 17:0/20:4 PI5P, 17:0/20:4 PI, 1-stearoyl-2- (5Z, 8Z, 11Z, 14Z-eicosatetraenoyl)-*sn*-glycero-3-phospho-(1-myo-inositol-3,4,5-trisphosphate) [18:0/20:4 PI(3,4,5)P_3_], 18:0/20:4 PI(3,4)P_2_, 18:0/20:4 PI(3,5)P_2_, 18:0/20:4 PI(4,5)P_2_, 18:0/20:4 PI3P, 18:0/20:4 PI4P, 18:0/20:4 PI5P, 18:0/20:4 PI, and 1-heptadecanoyl-2- (5Z, 8Z, 11Z, 14Z-eicosatetraenoyl)-*sn*-glycero-3-phospho-l-serine were purchased from Avanti. PI(4,5)P_2_ (8:0/8:0) and copanlisib was from Cayman. Methanol, chloroform, acetonitrile, ammonium acetate, ultrapure water, hydrochloric acid, 28% aqueous ammonia, and glacial acetic acid were all HPLC grade or MS grade and purchased from Fujifilm Wako Pure Chemical. Trimethylsilyl diazomethane was from Tokyo Kasei. DEAE Sepharose Fast Flow was from GE Healthcare. Glass tubes (12 mL) were from Corning. DMEM and bovine serum albumin (fatty acid-free) were from Nacalai Tesque. Hydrogen peroxide solution (30%) was from Fujifilm Wako. Chiral HPLC columns were from DAICEL and C18 columns were from GL Sciences. Recombinant human EGF was from PeproTech. Lipopolysaccharide (LPS; Escherichia coli O111: B4) was from Sigma-Aldrich. PIK-III was from Selleck Chemicals.

### Mice

All mice were under a normal light/dark condition (12 h light/12 h dark cycle). The animal colony was kept at 20–26 °C with 40–60 °C humidity. The generation and genotyping of *PbCre4-Pten*^*flox/flox*^ mice have been previously described^19,20^. Wild-type (WT) C57BL/6 J mice were obtained from CLEA Japan. All experimental protocols were reviewed and approved by the Akita University Institutional Committee for Animal Studies and the Tokyo Medical and Dental University Ethics Committee for Animal Experiments, and all experiments were performed according to their regulations.

### Plasmids

EGFP-tagged IpgD in the pEGFP-C1 expression vector was the generous gift of Dr. B. Payrastre (French Institute of Health and Medical Research). FLAG-tagged PIK3CA-E545K and PIK3CA-H1047R in the pcDNA3.1 expression vector were the kind gifts of Dr. S. Takasuga (Akita University). Plasmids were amplified in DH5a competent cells, and all inserts were confirmed by sequencing.

### Cell culture and transfection

HEK293T, PC3, LNCap and HeLa cells (ATCC) were maintained in DMEM (high glucose, Nacalai Tesque) supplemented with 0.1 mg/mL streptomycin, 100 units/mL penicillin, and 10% fetal bovine serum (FBS; Hyclone) at 37 °C (5% CO_2_, humidified atmosphere). HEK293T cells (3 × 10^6^) seeded in 100 mm dishes were transfected in 8 mL Opti-MEM (Gibco) using 10 µg plasmid DNA and 30 µL PEI Max (Polysciences) according to the manufacturer’s instructions. After 4 h of transfection, the medium was changed to fresh DMEM containing 10% FBS.

### Cell and tissue sample preparation

Cells were harvested using a cell scraper and collected into phosphate-buffered saline (PBS; 137 mM NaCl, 10 mM Na_2_HPO_4_, 2.7 mM KCl, 1.8 mM KH_2_PO_4_, pH7.4) transferred to a glass tube, and centrifuged at 1000×*g* for 5 min to pellet cells. Cells (3 × 10^6^) were resuspended in 1.5 mL methanol and subjected to lipid extraction (see below). For mouse prostate tissues, 20 mg tissue was transferred to a Lysing Matrix D tube (MP Bio) in 200 µl methanol. The tissue was homogenized using a FastPrep-24TM 5 G (MP Bio), transferred to a glass tube containing 1.5 mL methanol, and subjected to lipid extraction.

### Preparation of conditioned medium

Cells were grown to 60–70% confluence, whereupon the culture medium was replaced with serum-free medium (DMEM, 0.1% BSA, 10 mM HEPES, pH7.4). After 20 h incubation, culture supernatants were collected in 15 mL tubes, centrifuged at 5000×*g* for 5 min, and filtered through 0.45 µm cellulose acetate disks (DISMIC) to remove debris. The filtrate (conditioned medium; CM) was frozen in liquid nitrogen and stored at −80 °C. For analysis of extracellular phosphoinositides, filtrate (1 mL) was carefully thawed, mixed with 1.5 mL methanol, and subjected to lipid extraction.

### Preparation of mouse plasma

Plasma was prepared from the blood of anesthetized mice according to a standard protocol^23^. Briefly, C57BL/6 J mice were fasted for 6 h followed by intraperitoneal injection of PBS (control) or 50 mg/kg lipopolysaccharide (LPS). Blood was collected at 2 h post-injection and plasma was obtained by centrifugation at 6000×*g* for 10 min. Plasma (100 µL) was mixed with 1.5 mL methanol and subjected to lipid extraction.

### Preparation of exosomes

Cells (5 × 10^6^) were seeded in 150-mm culture dishes and cultured for 24 h. After two washes of the dishes with PBS, RPMI containing 1% exosome-depleted FBS was added. After 48 h incubation, culture supernatants were centrifuged at 2000×*g* for 10 min at 4 °C and then passed through a 0.22-μm filter (Millipore., Bedford, MA, USA) to remove cells and cellular debris. Filtered supernatants were ultracentrifuged at 110,000×*g* for 70 min at 4 °C (SW41Ti rotor, Beckman Coulter), and the pellets were washed with trehalose-containing HEPES buffer (20 mM HEPES pH7.4, 25 mM trehalose) to prevent the aggregation of exosomes. Resuspended pellets were re-ultracentrifuged at 110,000×*g* for 70 min at 4 °C, and finally resuspended in HEPES buffer (20 mM HEPES, pH7.4). The size distribution and concentration of exosomes were determined by nanoparticle tracking analysis (NTA) performed using a NanoSight LM10 instrument (Malvern, Worcestershire, UK). Exosomes (1 × 10^10^ particles) were mixed with 1.5 mL methanol and subjected to lipid extraction.

### Lipid extraction

A given biological sample in 1.5 mL methanol prepared as described above was mixed with 50 µL of a methanol/chloroform (9/1) solution containing 1 nmol C8:0/C8:0 PI(4,5)P_2_ (as an absorption inhibitor) and 10 pmol each of synthetic C17:0/C20:4 phosphoinositides [PI(3,4,5)P_3_, PI(3,4)P_2_, PI(3,5)P_2_, PI(4,5)P_2_, PI3P, PI4P, PI5P, and PI] as internal standards. Ultrapure water (750 µL), 2 M HCl (750 µL), and 1 M NaCl (200 µL) were added to this mixture. After vigorous vortex-mixing, 3 mL CHCl_3_ was added followed by further vortexing for 2 min. After centrifugation at 1200×*g* for 4 min at room temperature, the lower organic phase (crude lipid extract) was collected and transferred to a new glass tube.

### Pre-concentration of phosphoinositides

DEAE Sepharose Fast Flow (10% slurry) was rinsed twice with an equal volume of water, once with 1 M HCl, twice with water, once with 1 M NaOH, and twice with water. The resin was resuspended in methanol (50% slurry) and a 0.5 mL bed volume was packed in a Pasteur pipette (IWAKI) plugged with glass wool. The column was then equilibrated with chloroform. Crude lipid extract (2.9 mL) was mixed with 1.5 mL methanol and applied to the column, washed with 3 mL chloroform/methanol (1/1) and 2 mL chloroform/methanol/28% aqueous ammonia/glacial acetic acid (200/100/3/0.9), followed by elution with 1.5 mL chloroform/methanol/12 M hydrochloric acid/ultrapure water (12/12/1/1). The eluate was mixed with 850 µL 120 mM NaCl, centrifuged at 1200×*g* for 4 min at room temperature, and the resultant lower phase (purified phosphoinositides) was collected into a fresh glass tube.

### Methylation reaction

Purified phosphoinositides were derivatized by methylation using the method of Clark et al.^7^. Briefly, 150 µL 0.6 M trimethylsilyl diazomethane was added to the purified phosphoinositide fraction prepared as above at room temperature. After 10 min, the reaction was quenched with 20 µL of glacial acetic acid. The samples were mixed with 700 µL methanol/ultrapure water/chloroform (48/47/3) followed by vortexing for 1 min. After centrifugation at 1200×*g* for 4 min, the lower phase was taken to dryness under a stream of nitrogen and redissolved in 100 µL acetonitrile.

### Chiral column chromatography and tandem mass spectrometry

PRMC-MS was performed using a triple quadruple mass spectrometer QTRAP6500 (ABSciex) and a Nexera X2 HPLC system (Shimadzu) combined with a PAL HTC-xt (CTC Analytics) autosampler. The mass range of the instrument was set at *m/z* 5–2000. Spectra were recorded in the positive ion mode as [M + NH_4_]^+^ ions, and the scan duration of MS and MS/MS was 0.5 sec. The ion spray voltage was set at 5.5 kV, cone voltage at 30 V, and source block temperature at 100 °C. Curtain gas was set at 20 psi, collision gas at 9 psi, ion source gas pressures 1/2 at 50 psi, declustering potential at 100 V, entrance potential at 10 V, and collision cell exit potential at 12 V. Collision energy values for each acyl variant are listed in Supplementary Table 1. Lipid sample (10 µL) was injected using the autosampler, and molecules were separated using a CHIRALPAK IC-3 column [cellulose tris(3,5-dichlorophenylcarbamate), 2.1 mmφ × 250 mm, 3 mm, DAICEL] at 22 °C. LC was operated at a flow rate of 100 µL/min with a gradient as follows: 40% mobile phase A (methanol/5 mM ammonium acetate) and 60% mobile phase B (acetonitrile/5 mM ammonium acetate) were held for 1 min, linearly increased to 85% mobile phase A over 2 min, and held at 85% mobile phase A for 11 min. The column was re-equilibrated to 40% mobile phase A for 6 min before the next injection. Analyst 1.6.3 (SCIEX) was used for data acquisition and processing. MultiQuant (SCIEX) was used for manual data evaluation for peak integration. No background subtraction was performed. Gaussian smoothing width was 1.0 points. For quality control, the peaks from a sample run in which the cps of the surrogate internal standards (SIS; C37:4 phosphoinositides) from the MRM scan below 2 × 10^4^ were not subjected to quantification analysis. The sample peak area value (equivalent to 1 × 10^6^ cells, 10 mg tissue, 1 × 10^10^ exosome particles, or 1 ml plasma) was divided by the corresponding SIS peak area value (equivalent to 1 pmol) for relative quantification.

### Reverse-phase column chromatography-tandem mass spectrometry

C-MS/MS was performed with an LC system [UltiMate 3000 (Thermo Fisher Scientific) and HTC PAL autosampler (CTC Analytics)] connected to a triple-stage quadrupole mass spectrometer (TSQ Vantage, Thermo Fisher) as described previously^24^. Briefly, methylated lipids were separated on a C18 column (GL Sciences) using a solvent gradient as follows: 0–1 min hold 70% A/30% B, 1–3 min constant gradient to reach 90% A/10% B, 3–7.5 min constant at 90% A/10% B, 7.5–13 min 30% A/70% B, where mobile phase A was acetonitrile/water/70% ethylamine (800:200:1.3) and mobile phase B was acetonitrile/isopropanol/ 70% ethylamine (200:800:1.3). Multiple reaction monitoring (MRM) was employed in positive ion mode.

### Statistics

Values in each group are shown by mean ± SD, and the data are also plotted as symbols. Statistical differences between control and test groups were evaluated by the Dunnett’s test (Figs. 2c and 4a) or the unpaired Welch’s *t*-test (Figs. 2b, 3a, b, and 4e) using GraphPad Prism (GraphPad Software Inc.).

### Reporting summary

Further information on research design is available in the Nature Research Reporting Summary linked to this article.

## Supplementary information

Supplementary Information

Reporting Summary

## Data Availability

The mass spectrometry data generated in this study have been deposited in the MetaboBank and the MetaboLights databases under accession codes MTBKS201 and MTBLS3180, respectively. Source data are provided with this paper.

## Source data

Source Data File
