# Supplementary material for: A mass spectrometric method for in-depth profiling of phosphoinositide regioisomers and their disease-associated regulation
Source: Nat Commun. 2022 Jan 10;13:83. doi: 10.1038/s41467-021-27648-z (PMC8749000; doi:10.1038/s41467-021-27648-z)
Supplement: Supplementary file 1 — Supplementary Information [file 41467_2021_27648_MOESM1_ESM.pdf]

Supplementary Information for:

A mass spectrometric method for in-depth profiling phosphoinositide regioisomers and their disease-associated regulation

by Shin Morioka, Hiroki Nakanishi, Toshiyoshi Yamamoto, Junya Hasegawa, Emi Tokuda, Tomoya Hikita, Tomoko Sakihara, Yuuki Kugii, Chitose Oneyama, Masakazu Yamazaki, Akira Suzuki, Junko Sasaki and Takehiko Sasaki

This Supplementary Information section includes:

Supplementary Figures 1-5 and legends

Supplementary Tables 1-3

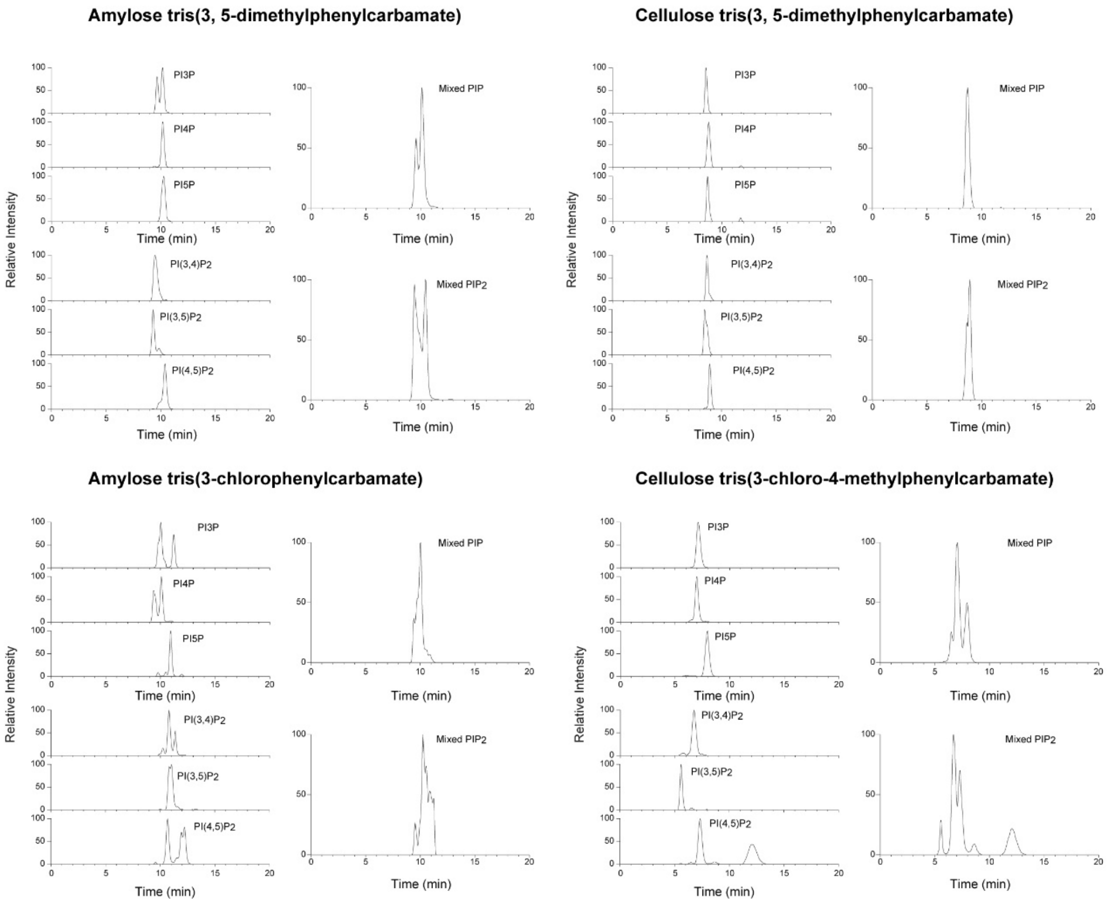

Figure S1

Supplementary Figure 1. Mass spectra obtained from chiral-HPLC-MS/MS analyses with four different chiral selectors. Standard C37:4 PIP and PIP_2 regioisomers were analyzed using the PRMC-MS protocol except that the indicated chiral selectors were used for HPLC separation. Due to inadequate retention time differences, none of these selectors enabled simultaneous and comprehensive measurement of regioisomers.

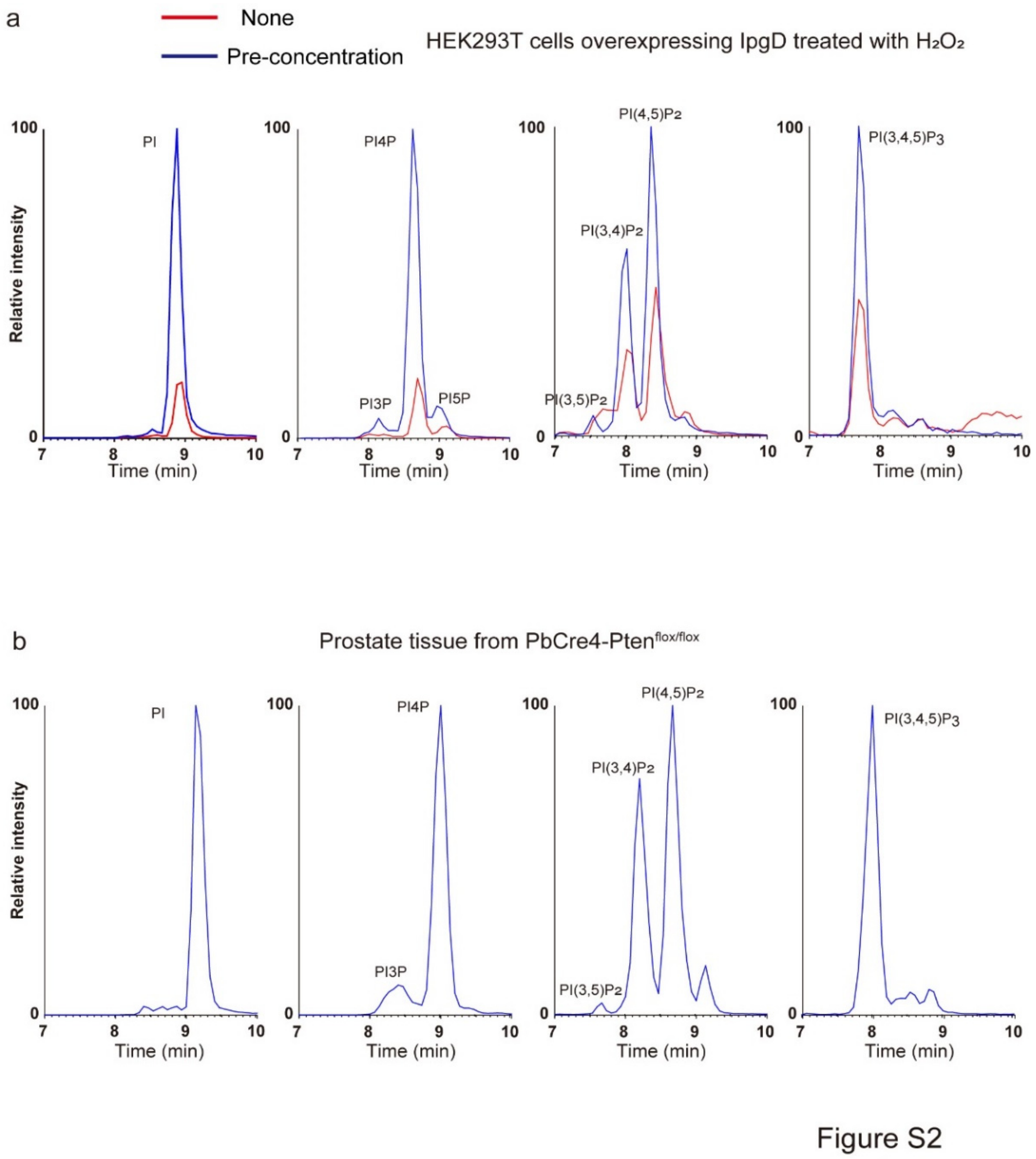

Supplementary Figure 2. Typical mass spectra from PRMC-MS analysis of biological samples.

a, Lipids were extracted from HEK293T cells that were transfected with an IpgD expression vector and then treated with 10 mM hydrogen peroxide for 2 min. Lipid extracts were processed either with (blue lines) or without (red lines) pre-concentration using DEAE Sepharose and analyzed with PRMC-MS. Chromatograms of C38:4 PI, PIP, PIP_2, and PIP_3 are shown. Note that pre-concentration increases the sensitivity of detection of each phosphoinositide variant by PRMC-MS. **b**, PRMC-MS chromatograms of C34:1 acyl variants of PI, PIP, PIP_2, and PIP_3 in a prostate tissue sample from a *PbCre4-Pten*$^{flox/flox}$ mouse.

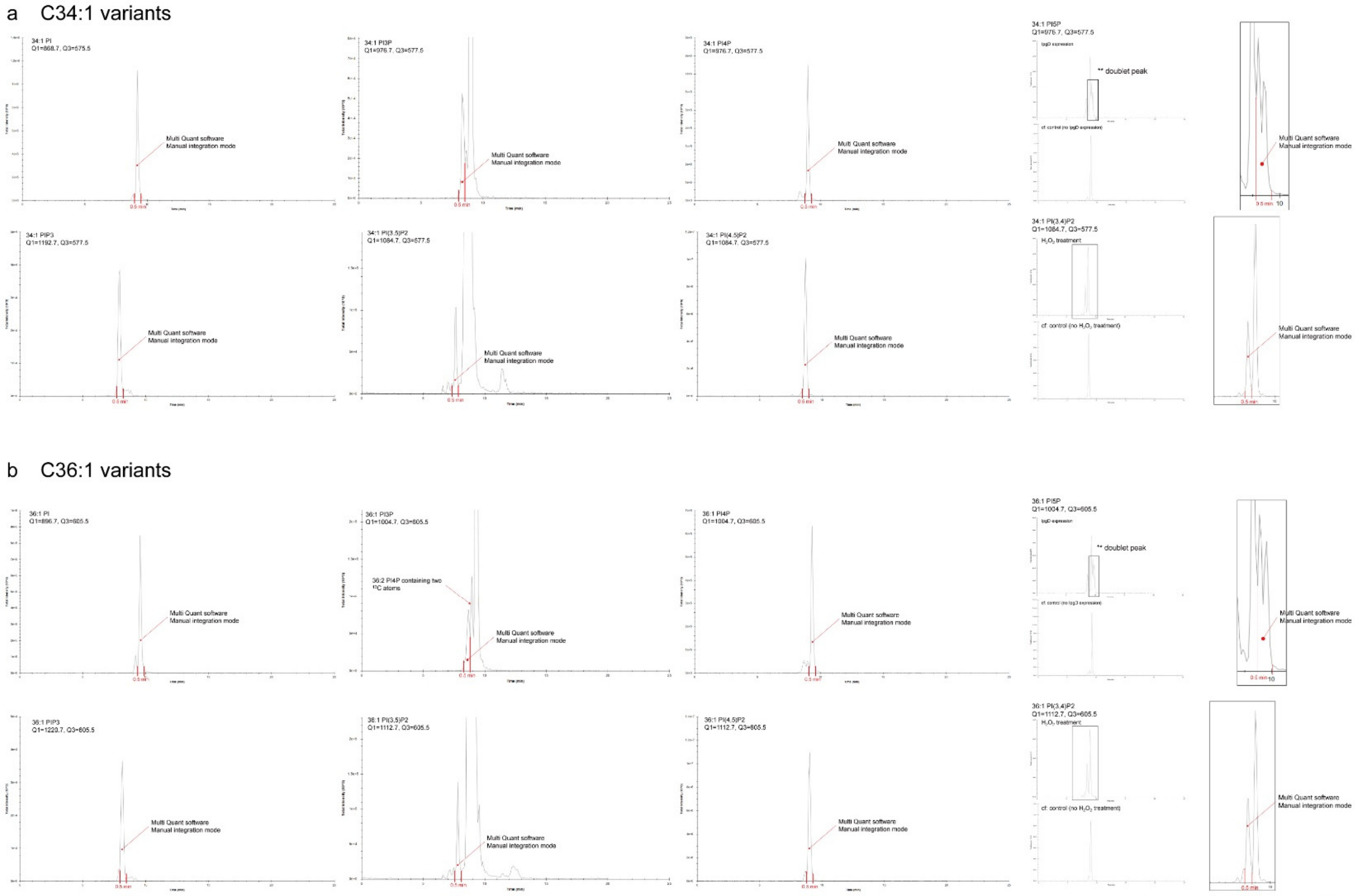
a C34:1 variants
34:1 PI
Q1=868.7, Q3=575.5
34:1 PI3P
Q1=976.7, Q3=577.5
34:1 PI4P
Q1=976.7, Q3=577.5
34:1 PI5P
Q1=976.7, Q3=577.5
** doublet peak
34:1 PIP3
Q1=1192.7, Q3=577.5
34:1 PI(3,5)P2
Q1=1084.7, Q3=577.5
34:1 PI(4,5)P2
Q1=1084.7, Q3=577.5
34:1 PI(3,4)P2
Q1=1084.7, Q3=577.5
Multi Quant software
Manual integration mode
0.5 min
b C36:1 variants
36:1 PI
Q1=896.7, Q3=605.5
36:1 PI3P
Q1=1004.7, Q3=605.5
36:2 PI4P containing two 13C atoms
36:1 PI4P
Q1=1004.7, Q3=605.5
36:1 PI5P
Q1=1004.7, Q3=605.5
** doublet peak
36:1 PIP3
Q1=1220.7, Q3=605.5
36:1 PI(3,5)P2
Q1=1112.7, Q3=605.5
36:1 PI(4,5)P2
Q1=1112.7, Q3=605.5
36:1 PI(3,4)P2
Q1=1112.7, Q3=605.5
Multi Quant software
Manual integration mode
0.5 min

Figure S3

c C36:2 variants

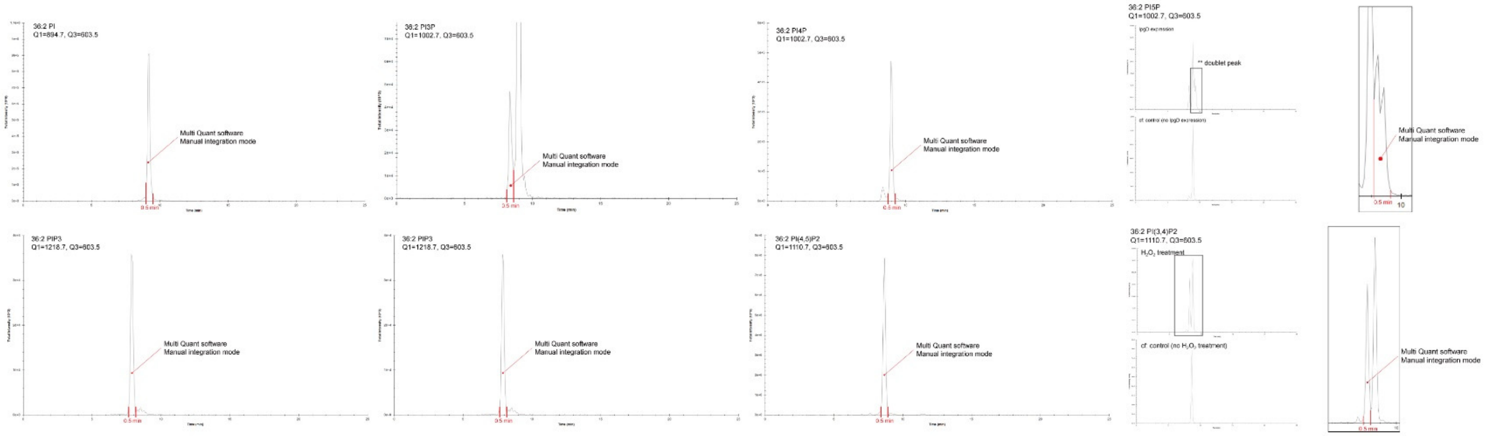

d C38:3 variants

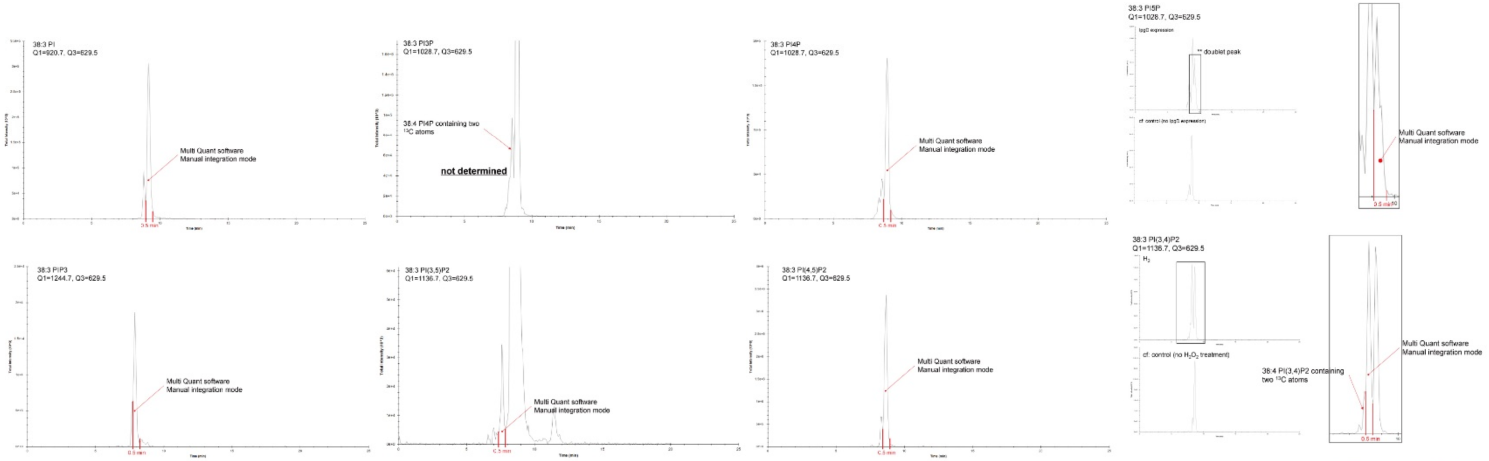

Figure S3

e C38:4 variants

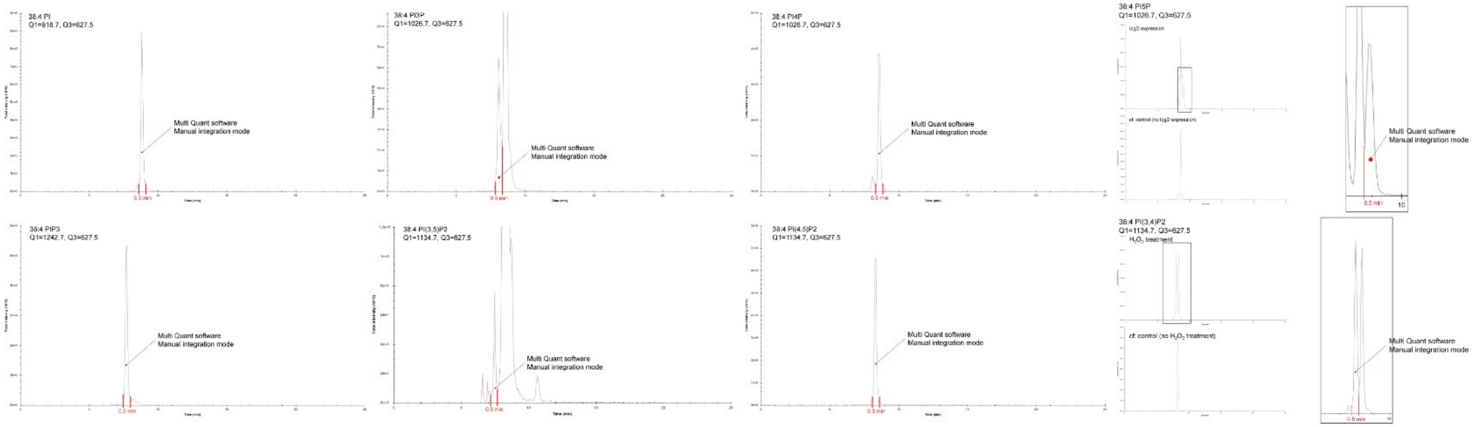

f C40:6 variants

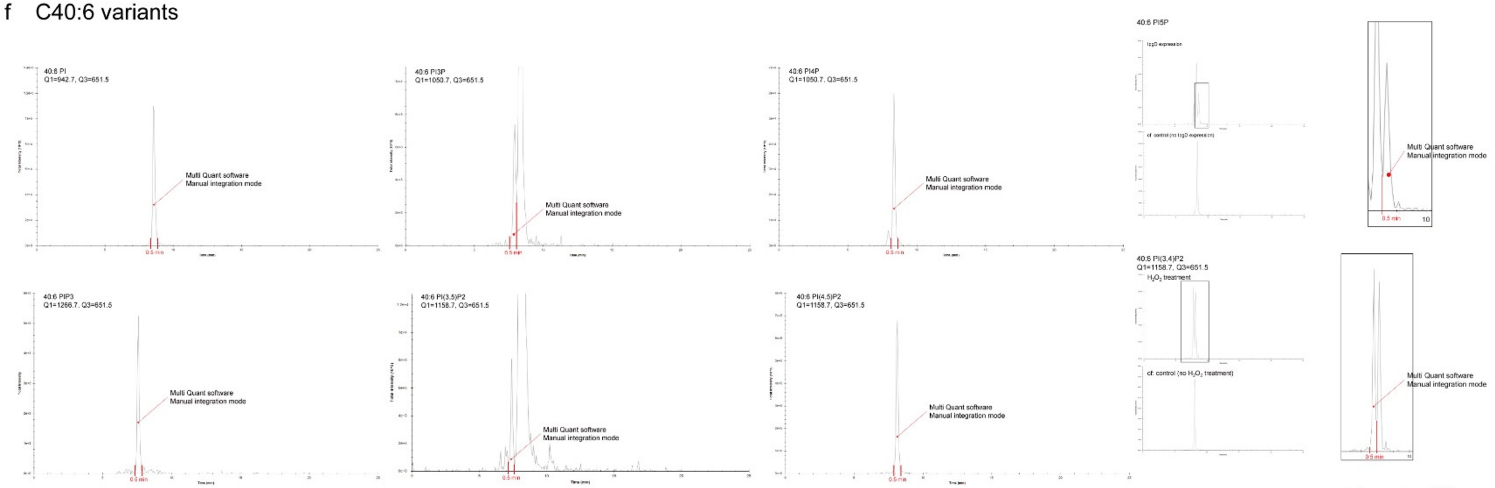

Figure S3

Supplementary Figure 3. Representative peak integration profiles. Chromatograms were obtained from PRMC-MS analysis of HEK293T cells. Analyst 1.6.3 (SCIEX) was used for data acquisition and processing. MultiQuant (SCIEX) was used for manual data evaluation for peak integration. No background subtraction was performed. Gaussian smoothing width was 1.0 points. For quality control, the peaks from a sample run in which the cps of the surrogate internal standards (C37:4 phosphoinositides) from the MRM scan below 2 x 10^4 were not subjected to quantification analysis. Note the occurrence of peaks corresponding to PI5P and PI(3,4)P_2 in response to IpgD expression and hydrogen peroxide treatment, respectively.

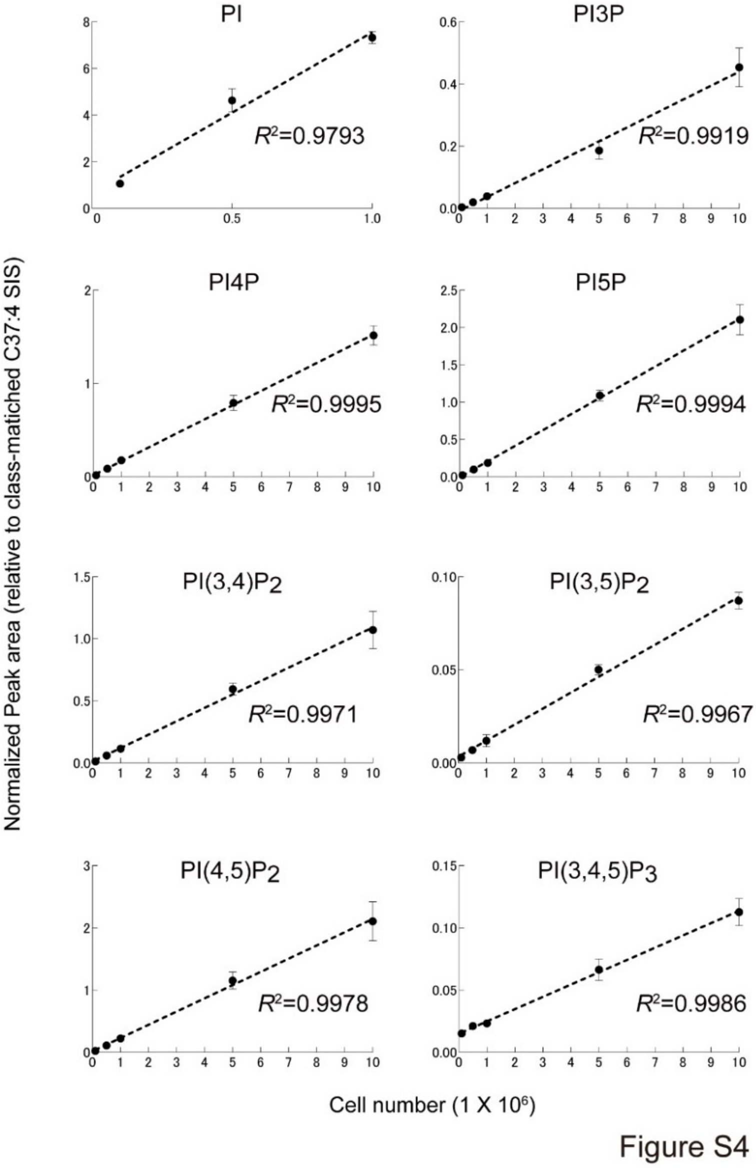

Supplementary Figure 4. Linear correlation between cell number and peak area of C38:4 phosphoinositides. Linear regression determination of cell number vs. peak area for the C38:4 acyl variant of each of the eight phosphoinositide classes. Phosphoinositides in 0.1, 0.5, 1, 5, or 10 x 10^6 HEK293T cells (n=3) were measured by PRMC-MS. Cells that either overexpressed IpgD or were treated with hydrogen peroxide were used to measure PI(5)P or $PI(3,4)P_2$, respectively. The same linear regression analysis was performed for another 113 acyl variants using the Graph Pad Prism (GraphPad Software Inc.), and these results are summarized in Table S3. R^2, correlation coefficient. Data are the mean ± SD (n=3 biologically independent samples). Source data are provided as a Source Data file.

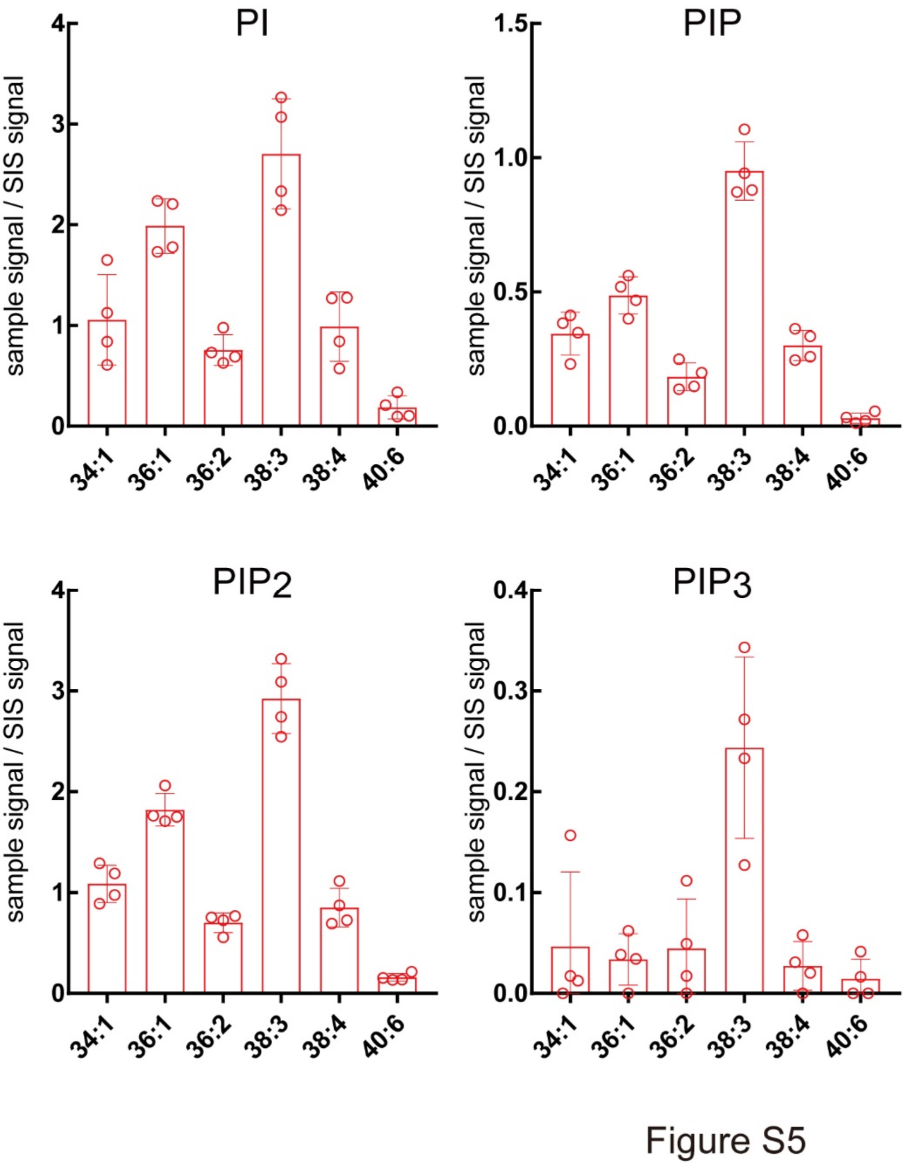

Supplementary Figure 5. Exosomal phosphoinositides examined by reverse-phase chromatography-mass spectrometry. Quantitation of the indicated phosphoinositides in exosome preparations. Lipids were separated by a C18 reverse-phase column and analyzed for the indicated acyl variants of PI, PIP, PIP_2, and PIP_3 using a triple quadrupole mass spectrometer and the same MRM method. This method does not distinguish between regioisomers of PIP and PIP_2. Data are the mean ± SD (n=4 biologically independent samples). Source data are provided as a Source Data file.

Supplementary Table 1. Calibration regression lines for synthetic phosphoinositide standards.

Phosphoinositide	R^2 value (2-100 fmol)	R^2 value (2 fmol-50 pmol)	LOD (fmol)	LOQ (fmol)	CV (%) (n=50)
C37:4 PI	0.9997	0.9957	2.45	7.42	11.3
C37:4 PI3P	0.9979	0.9969	6.43	19.5	8.4
C37:4 PI4P	0.9971	0.9858	7.52	22.8	12.0
C37:4 PI5P	0.9982	0.9935	6.01	18.2	10.2
C37:4 PI(3,4)P_2	0.9964	0.9990	8.46	25.6	9.1
C37:4 PI(3,5)P_2	0.9987	0.9955	5.00	15.2	10.6
C37:4 PI(4,5)P_2	0.9993	0.9995	3.60	10.9	8.5
C37:4 PI(3,4,5)P_3	0.9983	0.9966	5.85	17.7	11.4
C38:4 PI	0.9843	0.9898	17.70	53.26	n.d.
C38:4 PI3P	0.9936	0.9992	11.27	34.16	n.d.
C38:4 PI4P	0.9935	0.9950	11.29	34.20	n.d.
C38:4 PI5P	0.9975	0.9997	7.00	21.20	n.d.
C38:4 PI(3,4)P_2	0.9992	0.9995	4.00	12.12	5.7
C38:4 PI(3,5)P_2	0.9996	0.9977	2.88	8.73	4.9
C38:4 PI(4,5)P_2	0.9997	0.9995	2.44	7.41	5.6
C38:4 PI(3,4,5)P_3	0.9999	0.9974	1.30	3.94	5.2

R^2, correlation coefficient; LOD, lower limit of detection; LOQ, lower limit of quantification; CV, coefficient of variation. LOD and LOQ were determined by the calibration curve method. Average peak area of each analyte was plotted against concentration (2, 5, 10, 20, 50 and 100 fmol). LOD and LOQ were expressed as 3.3 × Syx/a and 10.0 × Syx/a, respectively, where Syx is residual variance due to regression

and “a” is the mean slope of the linear regression curves obtained by GraphPad Prism (GraphPad software Inc.). n.d., not determined. Source data are provided as a Source Data file.

Supplementary Table 2. MRM transitions and collision energy values used to measure each phosphoinositide acyl variant.

Acyl variant	Q1 (parent *m/z*)	Q3 (fragment *m/z*)	Collision energy (eV)
32:1 PI	840.7	549.5	32
32:0 PI	842.7	551.5	32
34:2 PI	866.7	575.5	32
34:1 PI	868.7	577.5	32
34:0 PI	870.7	579.5	32
36:4 PI	890.7	599.5	33
36:3 PI	892.7	601.5	33
36:2 PI	894.7	603.5	33
36:1 PI	896.7	605.5	33
36:0 PI	898.7	607.5	33
37:4 PI	904.7	613.5	34
38:6 PI	914.7	623.5	34
38:5 PI	916.7	625.5	34
38:4 PI	918.7	627.5	34
38:3 PI	920.7	629.5	34
40:6 PI	942.7	651.5	35
40:5 PI	944.7	653.5	35
40:4 PI	946.7	655.5	35
32:1 PI3P/PI4P/PI5P	948.7	549.5	35
32:0 PI3P/PI4P/PI5P	950.7	551.5	35
34:2 PI3P/PI4P/PI5P	974.7	575.5	35
34:1 PI3P/PI4P/PI5P	976.7	577.5	35
34:0 PI3P/PI4P/PI5P	978.7	579.5	35
36:4 PI3P/PI4P/PI5P	998.7	599.5	36
36:3 PI3P/PI4P/PI5P	1000.7	601.5	36
36:2 PI3P/PI4P/PI5P	1002.7	603.5	36
36:1 PI3P/PI4P/PI5P	1004.7	605.5	36
36:0 PI3P/PI4P/PI5P	1006.7	607.5	36
37:4 PI3P/PI4P/PI5P	1012.7	613.5	37
38:6 PI3P/PI4P/PI5P	1022.7	623.5	37
38:5 PI3P/PI4P/PI5P	1024.7	625.5	37

38:4 PI3P/PI4P/PI5P	1026.7	627.5	37
38:3 PI3P/PI4P/PI5P	1028.7	629.5	37
40:6 PI3P/PI4P/PI5P	1050.7	651.5	38
40:5 PI3P/PI4P/PI5P	1052.7	653.5	38
40:4 PI3P/PI4P/PI5P	1054.7	655.5	38
32:1 PI(3,4)P_2/PI(3,5)P_2/PI(4,5)P_2	1056.7	549.5	36
32:0 PI(3,4)P_2/PI(3,5)P_2/PI(4,5)P_2	1058.7	551.5	36
34:2 PI(3,4)P_2/PI(3,5)P_2/PI(4,5)P_2	1082.7	575.5	36
34:1 PI(3,4)P_2/PI(3,5)P_2/PI(4,5)P_2	1084.7	577.5	36
34:0 PI(3,4)P_2/PI(3,5)P_2/PI(4,5)P_2	1086.7	579.5	36
36:4 PI(3,4)P_2/PI(3,5)P_2/PI(4,5)P_2	1106.7	599.5	37
36:3 PI(3,4)P_2/PI(3,5)P_2/PI(4,5)P_2	1108.7	601.5	37
36:2 PI(3,4)P_2/PI(3,5)P_2/PI(4,5)P_2	1110.7	603.5	37
36:1 PI(3,4)P_2/PI(3,5)P_2/PI(4,5)P_2	1112.7	605.5	37
36:0 PI(3,4)P_2/PI(3,5)P_2/PI(4,5)P_2	1114.7	607.5	37
37:4 PI(3,4)P_2/PI(3,5)P_2/PI(4,5)P_2	1120.7	613.5	38
38:6 PI(3,4)P_2/PI(3,5)P_2/PI(4,5)P_2	1130.7	623.5	38
38:5 PI(3,4)P_2/PI(3,5)P_2/PI(4,5)P_2	1132.7	625.5	38
38:4 PI(3,4)P_2/PI(3,5)P_2/PI(4,5)P_2	1134.7	627.5	38
38:3 PI(3,4)P_2/PI(3,5)P_2/PI(4,5)P_2	1136.7	629.5	38
40:6 PI(3,4)P_2/PI(3,5)P_2/PI(4,5)P_2	1158.7	651.5	39
40:5 PI(3,4)P_2/PI(3,5)P_2/PI(4,5)P_2	1160.7	653.5	39
40:4 PI(3,4)P_2/PI(3,5)P_2/PI(4,5)P_2	1162.7	655.5	39
32:1 PI(3,4,5)P_3	1164.7	549.5	38
32:0 PI(3,4,5)P_3	1166.7	551.5	38
34:2 PI(3,4,5)P_3	1190.7	575.5	38
34:1 PI(3,4,5)P_3	1192.7	577.5	38
34:0 PI(3,4,5)P_3	1194.7	579.5	38
36:4 PI(3,4,5)P_3	1214.7	599.5	39
36:3 PI(3,4,5)P_3	1216.7	601.5	39
36:2 PI(3,4,5)P_3	1218.7	603.5	39
36:1 PI(3,4,5)P_3	1220.7	605.5	39
36:0 PI(3,4,5)P_3	1222.7	607.5	39
37:4 PI(3,4,5)P_3	1228.7	613.5	40
38:6 PI(3,4,5)P_3	1238.7	623.5	40
38:5 PI(3,4,5)P_3	1240.7	625.5	40

38:4 PI(3,4,5)P_3	1242.7	627.5	40
38:3 PI(3,4,5)P_3	1244.7	629.5	40
40:6 PI(3,4,5)P_3	1266.7	651.5	41
40:5 PI(3,4,5)P_3	1268.7	653.5	41
40:4 PI(3,4,5)P_3	1270.7	655.5	41

Supplementary Table 3. Linear dynamic ranges for specific phosphoinositide acyl variants.

Calibration ranges that gave the correlation coefficient (R^2) between cell number and phosphoinositide quantity listed was 0.1-1 x 10^6 cells for PI and 0.1-10 x 10^6 cells for other classes. Also see the linear regression lines for the C38:4 phosphoinositides in Figure S4. N.A., not available (due to incomplete peak separation and poor reproducibility). Source data are provided as a Source Data file.

	PI	PI3P	PI4P	PI5P	PI(3,4)P_2	PI(3,5)P_2	PI(4,5)P_2	PI(3,4,5)P_3
C32:0	0.9782	N.A.	0.9905	0.9998	0.9986	0.9608	0.9878	0.9953
C32:1	0.9766	0.9823	0.9970	0.9996	0.9991	0.9986	0.9929	0.9802
C34:0	0.8674	N.A.	0.9760	N.A.	N.A.	0.9477	0.9903	N.A.
C34:1	0.9709	0.9909	0.9939	0.9999	0.9993	0.9849	0.9856	0.9955
C34:2	0.9884	0.9866	0.9908	0.9988	0.9994	0.9999	0.9979	0.9984
C36:1	0.9430	0.9890	0.9947	0.9980	0.9999	0.9870	0.9958	0.9912
C36:2	0.9911	0.9925	0.9976	0.9998	0.9996	0.9957	0.9951	0.9915
C36:3	0.9899	0.9688	0.9993	0.9991	0.9994	0.9924	0.9984	0.9947
C36:4	0.9894	0.9817	0.9935	0.9931	0.9998	0.9731	0.9964	0.9598
C38:3	0.9700	N.A.	0.9977	0.9999	0.9981	0.9958	0.9956	0.9885
C38:4	0.9793	0.9919	0.9995	0.9994	0.9971	0.9967	0.9978	0.9986
C38:5	0.9922	0.9801	0.9993	0.9968	0.9987	0.9477	0.9918	0.9973
C38:6	0.9956	0.9925	0.9967	0.9658	0.9978	0.9533	0.9967	0.9879
C40:4	0.9783	N.A.	0.9931	0.9999	0.9865	0.9686	0.9939	0.9650
C40:5	0.9856	N.A.	0.9995	0.9993	0.9955	0.9937	0.9950	0.9821
C40:6	0.9826	0.9768	0.9990	0.9991	0.9969	0.9909	0.9972	0.9986
