# Supplementary material for: A mass spectrometric method for in-depth profiling of phosphoinositide regioisomers and their disease-associated regulation
Source: Nat Commun. 2022 Jan 10;13:83. doi: 10.1038/s41467-021-27648-z (PMC8749000; doi:10.1038/s41467-021-27648-z)
Supplement: Supplementary file 2 — Reporting Summary [file 41467_2021_27648_MOESM2_ESM.pdf]

nature portfolio

Corresponding author(s): Takehiko Sasaki and Junko Sasaki

Last updated by author(s): Nov 4, 2021

# Reporting Summary

Nature Portfolio wishes to improve the reproducibility of the work that we publish. This form provides structure for consistency and transparency in reporting. For further information on Nature Portfolio policies, see our Editorial Policies and the Editorial Policy Checklist.

## Statistics

For all statistical analyses, confirm that the following items are present in the figure legend, table legend, main text, or Methods section.

| n/a | Confirmed | |
|---|---|---|
| ☐ | ☒ | The exact sample size ($n$) for each experimental group/condition, given as a discrete number and unit of measurement |
| ☐ | ☒ | A statement on whether measurements were taken from distinct samples or whether the same sample was measured repeatedly |
| ☐ | ☒ | The statistical test(s) used AND whether they are one- or two-sided<br>*Only common tests should be described solely by name; describe more complex techniques in the Methods section.* |
| ☐ | ☒ | A description of all covariates tested |
| ☐ | ☒ | A description of any assumptions or corrections, such as tests of normality and adjustment for multiple comparisons |
| ☐ | ☒ | A full description of the statistical parameters including central tendency (e.g. means) or other basic estimates (e.g. regression coefficient) AND variation (e.g. standard deviation) or associated estimates of uncertainty (e.g. confidence intervals) |
| ☐ | ☒ | For null hypothesis testing, the test statistic (e.g. $F$, $t$, $r$) with confidence intervals, effect sizes, degrees of freedom and $P$ value noted<br>*Give P values as exact values whenever suitable.* |
| ☒ | ☐ | For Bayesian analysis, information on the choice of priors and Markov chain Monte Carlo settings |
| ☒ | ☐ | For hierarchical and complex designs, identification of the appropriate level for tests and full reporting of outcomes |
| ☒ | ☐ | Estimates of effect sizes (e.g. Cohen's $d$, Pearson's $r$), indicating how they were calculated |

*Our web collection on statistics for biologists contains articles on many of the points above.*

## Software and code

Policy information about availability of computer code

| | |
|---|---|
| Data collection | MultiQuant 3.0.2 software (Sciex), Xcalibur 2.0 (Thermo Fisher Scientific), Analyst 1.6.3 (SCIEX) |
| Data analysis | GraphPad Prism version 7J (GraphPad Software Inc.) |

For manuscripts utilizing custom algorithms or software that are central to the research but not yet described in published literature, software must be made available to editors and reviewers. We strongly encourage code deposition in a community repository (e.g. GitHub). See the Nature Portfolio guidelines for submitting code & software for further information.

## Data

Policy information about availability of data

All manuscripts must include a data availability statement. This statement should provide the following information, where applicable:
- Accession codes, unique identifiers, or web links for publicly available datasets
- A description of any restrictions on data availability
- For clinical datasets or third party data, please ensure that the statement adheres to our policy

The mass spectrometry data generated in this study have been deposited in the MetaboBank and the MetaboLights databases under accession codes MTBKS201 [https://ddbj.nig.ac.jp/public/metabobank/study/MTBKS201] and MTBLS3180 [www.ebi.ac.uk/metabolights/MTBLS3180], respectively. Source data are provided with this paper.

# Field-specific reporting

Please select the one below that is the best fit for your research. If you are not sure, read the appropriate sections before making your selection.

- [x] Life sciences
- [ ] Behavioural & social sciences
- [ ] Ecological, evolutionary & environmental sciences

For a reference copy of the document with all sections, see nature.com/documents/nr-reporting-summary-flat.pdf

# Life sciences study design

All studies must disclose on these points even when the disclosure is negative.

| | |
|---|---|
| Sample size | No sample size calculations were performed. Sample size was chosen to obtain statistical significance values. |
| Data exclusions | Data were not excluded from the analyses performed in this study. |
| Replication | All experiments were performed independently at least three times. All attempts at replication were successful. |
| Randomization | For mouse experiments, littermate mice were allocated into experimental groups without bias, except that the mice were genotyped before use. For cell experiments, randomization was not relevant, because cells were treated in the same way by the same researchers regardless of the treatments. |
| Blinding | The investigators who performed lipid analyses were blinded to the genotype of mouse samples. The investigators were not blinded to the cellular and chemical samples because the same investigators prepared and executed lipid quantification of the samples. |

# Reporting for specific materials, systems and methods

We require information from authors about some types of materials, experimental systems and methods used in many studies. Here, indicate whether each material, system or method listed is relevant to your study. If you are not sure if a list item applies to your research, read the appropriate section before selecting a response.

## Materials & experimental systems

| n/a | Involved in the study | |
|---|---|---|
| x | | Antibodies |
| | x | Eukaryotic cell lines |
| x | | Palaeontology and archaeology |
| | x | Animals and other organisms |
| x | | Human research participants |
| x | | Clinical data |
| x | | Dual use research of concern |

## Methods

| n/a | Involved in the study | |
|---|---|---|
| x | | ChIP-seq |
| x | | Flow cytometry |
| x | | MRI-based neuroimaging |

## Eukaryotic cell lines

Policy information about cell lines

| | |
|---|---|
| Cell line source(s) | HEK293T, PC3, LNCap and HeLa cells were from ATCC. |
| Authentication | None of the cell lines used were authenticated in our lab. |
| Mycoplasma contamination | All cell lines tested negative for mycoplasma. |
| Commonly misidentified lines (See ICLAC register) | There were no commonly misidentified cell lines used in this study. |

## Animals and other organisms

Policy information about studies involving animals; ARRIVE guidelines recommended for reporting animal research

| | |
|---|---|
| Laboratory animals | All mice were under a normal light/dark condition (12 hrs light/12 hrs dark cycle). The animal colony was kept at 20-26 degree Celsius with 40-60% humidity. The generation and genotyping of PbCre4-Ptenflox/flox mice have been previously described. Wild type (WT) C57BL/6J mice were obtained from CLEA Japan. Male 12-week-old mice were used in this study. All experimental protocols were reviewed and approved by the Akita University Institutional Committee for Animal Studies and the Tokyo Medical and Dental University Ethics Committee for Animal Experiments, and all experiments were performed according to their regulations. |
| Wild animals | No wild animals were used in this study. |

| | |
|---|---|
| Field-collected samples | No field-collected samples were used in this study. |
| Ethics oversight | Experimental protocols were reviewed and approved by the Akita University Institutional Committee for Animal Studies and the Tokyo Medical and Dental University Ethics Committee for Animal Experiments, and all experiments were performed according to their regulations. |

Note that full information on the approval of the study protocol must also be provided in the manuscript.
